# Micro-Current Stimulation Can Modulate the Adipogenesis Process by Regulating the Insulin Signaling Pathway in 3T3-L1 Cells and *ob*/*ob* Mice

**DOI:** 10.3390/life13020404

**Published:** 2023-02-01

**Authors:** Hana Lee, Jin-Ho Lee, Doyong Kim, Donghyun Hwang, Minjoo Lee, Halim Chung, Tack-Joong Kim, Han Sung Kim

**Affiliations:** 1 Department of Biomedical Engineering, Yonsei University, Wonju 26493, Republic of Korea; 2 Division of Biological Science and Technology, Yonsei University, Wonju 26493, Republic of Korea

**Keywords:** obesity, adipogenesis, micro-current stimulation, insulin signaling pathway, 3T3-L1 cells, *ob*/*ob* mice model

## Abstract

Obesity is a disease in which fat is abnormally or excessively accumulated in the body, and many studies have been conducted to overcome it with various techniques. In this study, we evaluated whether micro-current stimulation (MCS) can be applied to prevent obesity by regulating the adipogenesis through 3T3-L1 cells and *ob*/*ob* mice. To specify the intensity of MCS, Oil Red O staining was conducted with various intensities of MCS. Based on these, subsequent experiments used 200 and 400 μA for the intensity of MCS. The expressions of insulin signaling pathway-related proteins, including phosphorylation of IGF-1 and IR, were decreased in all MCS groups, and in turn, downstream signals such as Akt and ERK were decreased. In addition, MCS reduced the nucleus translocation of PPAR-γ and decreased the protein expression of C/EBP-α. In the *ob*/*ob* mouse model, MCS reduced body weight gain and abdominal adipose tissue volume. In particular, the concentration of triglycerides in serum was also decreased. Taken together, our findings showed that MCS inhibited lipid accumulation by regulating insulin signaling in 3T3-L1, and it was effective at reducing body weight and adipose tissue volume in *ob*/*ob* mice. These suggest that MCS may be a useful treatment approach for obesity.

## 1. Introduction

Abnormal or excessive adipose tissue accumulation occurs when lipids accumulate in adipocytes due to an imbalance between intake and calorie expenditure, and these cause the risk of being overweight and obesity [1]. According to the World Health Organization in 2008, 1.4 billion adults were overweight, of which 500 million were obese [2]. Obesity causes several chronic, immune system disruptions, leading to insulin resistance and β-cell dysfunction [3]. Moreover, it increases the prevalence of type 2 diabetes mellitus (T2DM) and non-alcoholic fatty liver disease (NAFLD), and it causes abnormal glucose homeostasis, hypertension, metabolic and cardiovascular problems in the body [4]. Since obesity is directly related to various health problems, many studies have been conducted to inhibit adipogenesis through various approaches to reduce fat accumulation [5,6].

Adipogenesis is a multi-step process of the proliferation and differentiation of adipocyte precursor cells into mature adipocytes [7]. Adipose tissue expansion occurs through two modes: hyperplasia is an increase in the number of adipocytes, and hypertrophy means enlargement of adipocytes [8]. For this reason, a possible therapeutic approach to obesity would be to reduce the size or number of adipocytes by regulating adipogenesis. 

Adipogenesis is accomplished through mitotic clonal expansion (MCE), early differentiation, and terminal differentiation [9]. MCE is a prerequisite for the terminal differentiation of preadipocytes. In this process, growth-arrested cells re-enter the cell cycle, and cell numbers also increase before adipogenesis-related genes are expressed. It has been suggested that the MCE enables DNA remodeling for gene expression during adipogenesis [10,11]. In particular, the Ras/mitogen-activated protein kinase (MAPK) pathway is known to regulate cell growth and protein synthesis in adipogenesis [9]. This pathway, along with tyrosine phosphorylation of insulin receptor substrate (IRS) proteins, interacts with various proteins to activate Ras and stimulate the MEK/ERK cascade [12]. Activated AKT and phosphorylated ERK by tyrosine phosphorylation of IRS-1 were mainly involved in MCE initiation. Similarly, insulin receptor (IR) has also been reported as a common upstream regulator of ERK and Akt signaling [13]. In particular, tyrosine autophosphorylation of IR is the first cellular response to insulin stimulation and plays a pivotal role in phosphorylating substrates such as IRS-1 that serve as docking for downstream proteins [14]. Because insulin initiates proliferative signaling at the onset of MCE, IR and its downstream signals, Raf-MEK-ERK and IRS-PI3K-Akt signaling, play important roles in adipogenesis [15]. In addition, it is reported that knockout of IR is effective in protecting against obesity and obesity-related glucose intolerance in a mice model [16]. Therefore, the inhibition of ERK and Akt can be considered one effective strategy to inhibit the adipogenesis process.

In our previous study, it is reported that external electrical stimulation inhibits adipogenesis by activating Wnt/β-catenin signaling [17]. Although there are differences depending on the types of stimulation and cells, it has been reported that electrical stimulation affects various intracellular signaling cascades including PI3K, cAMP, PTEN, ERK1/2, and calcium signaling [18,19,20]. The electric activity of cells can regulate a variety of cell functions including growth, adhesion, differentiation, proliferation, activation of intracellular pathways, secretion of proteins and gene expression [21,22,23]. Due to the electrical properties of the cell, external electrical stimulation has been used to induce physiological changes in cells. However, studies examining whether electrical stimulation can modulate signals related to ERK and AKT, which are important in the process of adipogenesis, in preadipocytes have not been conducted.

Therefore, the purpose of this study was to identify the effects of electrical stimulation on the regulation of adipogenesis in 3T3-L1 cells through ERK, Akt and their related factors, and second, to determine whether electrical stimulation can alleviate obesity in *ob*/*ob* mice.

## 2. Materials and Methods

### 2.1. Materials

3T3-L1 preadipocytes were purchased from the American Type Culture Collection (ATCC; CL-173, Manassas, VA, USA). 3-Isobutyl-methylxanthine (IBMX), dexamethasone (DEX), insulin, Dulbecco’s modified Eagle’s medium (DMEM), penicillin–streptomycin (PS), bovine calf serum (BCS), fetal bovine serum (FBS), and Oil Red O staining solution were purchased from Sigma-Aldrich Chemical Co. (St. Louis, MO, USA). To measure cell viability, an EZ-Cytox cell viability assay kit was purchased from the Daeil Labservice (Seoul, Republic of Korea). A Triglyceride Quantification Assay Kit (Colorimetric/Fluorometric) was purchased from abcam (Cambridge, UK). Lysis buffer was purchased from iNtRON Biotechnology Inc. (Seongnam, Republic of Korea). Protease and Phosphatase Inhibitor Mini Tablets and a BCA assay kit were purchased from Thermo Scientific (Rockford, IL, USA).

### 2.2. Micro-Current Stimulation

The electrical stimulation used in this study is micro-current stimulation (MCS) with a current intensity of 1 mA or less. We self-developed the device to apply electrical stimulation to both cells and animals. For providing constant current to animal skin or cell media whose impedance changes continuously, an impedance feedback circuit was applied to this device. In the cell experiments, biphasic currents of 200 and 400 μA with a frequency of 10 Hz were applied for 60 min before differentiation induction. To apply the MCS to the animal model, an adhesive electrode made of conductive metal and hydrogel was attached to the upper and lower abdomen of animals, and then, biphasic currents of 200 and 400 μA with a frequency of 10 Hz were applied 20 min/a day, 5 days/a week, for 4 weeks.

### 2.3. 3T3-L1 Cell Culture and Differentiation

3T3-L1 preadipocytes were cultured in DMEM containing 10% BCS and 1% PS as growth medium at 37 °C in a humidified incubator containing 5% CO_2_. 3T3-L1 cells were seeded in 6-well plates at 2 × 10^5^ cells/well. MCS was applied when the cell confluency reached 100%, and then differentiation was induced. To induce the differentiation, two days after achieving confluency, the culture medium was replaced with differential medium containing 5 mM IBMX, 1 μM dexamethasone, and 10 μg/mL insulin (MDI) for 2 days. The culture medium was replaced with DMEM containing 10% FBS and insulin and then cultured for 48 h. The cells were cultured in DMEM, 10% FBS and 1% PS until most of the cells were differentiated into mature adipocytes.

### 2.4. Cell Viability

We evaluated the effect on MCS on cell viability depending on the intensity of MCS in 3T3-L1 cells. The intensities of MCS used in the experiment were 25, 50, 100, 200, and 400 μA. After each intensity of MCS was applied to 6 well plates at 100% confluency, colorimetric analysis was performed by WST-1 assay on the 6th day. Cells were incubated for 1 h, and the growth medium without WST solution was designated to blank. Cell viability was measured at 450 nm using a microplate reader (Epoch, BioTek Instruments, Winooski, VT, USA).

### 2.5. Oil Red O Staining

After the end of the differentiation process, the cells were washed twice with PBS and then incubated with 10% fresh formalin solution for 20 min at RT. After removing the 10% fresh formalin, the cells were treated with 60% isopropanol. Cells were stained with Oil Red O solution for 10 min at RT, after which excess Oil Red O solution was washed with distilled water. For quantification, Oil Red O contained in the cells was extracted with 100% isopropanol and determined with a microplate reader at 520 nm. The values were calculated as percentages of the control wells (% of control).

### 2.6. Confocal Microscopy

For confocal microscopy, 3T3-L1 cells were cultured in 35 mm dishes with a cover glass (Mattek Corp, Ashland, MA, USA), which was followed by cell differentiation, and each intensity of MCS was applied. The sample was permeabilized by 0.1% Triton X-100 in PBS for 1 min. The cells were placed in blocking solution (3% BSA in phosphate-buffered saline containing 0.1% Tween-20 (PBS-T)) for 30 min at RT. Cells were incubated with a 1:1000 dilution of PPAR-γ primary antibody (#2443, Cell Signaling Technology, Danvers, MA, USA) overnight at 4 °C. After washing twice, cells were incubated with 1:500 dilution of the Alexa Flour 488 (green, Invitrogen, CA, USA). In order to facilitate observation of the nucleus, cells were cultured for 10 min by immobilizing 3.7% paraformaldehyde at RT with fluorescent dye DAPI diluted in PBS-T. GFP images were obtained with an LSM710 confocal microscope (Carl Zeiss, Oberkochen, Germany). During confocal microscopic observation, all images were taken at the same setting. Images were measured at wavelengths of 495 and 519 nm. To observe the translocation of PPAR-γ, images were presented using the Zen lite software (Carl Zeiss, Oberkochen, Germany). The data were presented by four different kinds of image.

### 2.7. Immunoblotting

Cell lysates and epididymal fat lysates were prepared with lysis buffer containing protease inhibitor. The protein concentrations were quantified via a BCA assay kit, and 20 μg of protein was separated by SDS-PAGE gels and then transferred onto the PVDF membrane. The membranes were then blocked using 3% nonfat dry milk in Tris-buffered saline-Tween 20 (TBS-T) for 1 h and then probed overnight with the primary antibodies (1:1000) at 4 °C. In this experiment, we used primary antibodies including: p-IGF-1 receptor (#4568, Cell Signaling Technology), Phospho-Insulin Receptor β (Tyr1345) (#3026, Cell Signaling Technology), p-Akt (#9271, Cell Signaling Technology), Akt (#4691, Cell Signaling Technology), p-ERK1/2 (#4377, Cell Signaling Technology), ERK1/2 (#4695, Cell Signaling Technology), PPAR-γ (#2443, Cell Signaling Technology), C/EBP-α (#2295, Cell Signaling Technology) and β-actin (#4967, Cell Signaling Technology). The membranes were washed and incubated with anti-Rabbit IgG, HRP-linked Secondary Antibody (#7074, Cell Signaling Technology) for 2 h at RT, and specific protein bands were detected with Amersham™ ECL™ Prime Western Blotting Detection Reagent (RPN2236, GE Healthcare) and visualized on Image Quant LAS 500 or 4000 (GE healthcare, UK). Band intensities were quantified and calculated using Image J software (1.52a version, National Institutes of Health, Bethesda, MD, USA).

### 2.8. Animals

The protocols for all procedures were approved by the Yonsei University Animal Care Committee (YWCI-201704-007-01). Fifteen 6-week-old male *ob*/*ob* mice (38.23 ± 1.93 g) and five 6-week-old male C57BL/6J mice (21.88 ± 0.53 g) were used in this study. Five 6-week-old male C57BL/6J mice were allocated to the control group (CON, n = 5), and fifteen 6-week-old male *ob*/*ob* mice were randomly assigned to three groups: the obesity group (ob, n = 5), the obesity with 200 μA MCS group (200 μA, n = 5) and the obesity with 400 μA MCS group (400 μA, n = 5). All the animals were maintained under a 12:12 h light–dark cycle (23 ± 3 °C, 50 ± 10% humidity) with normal chow and water, ad libitum, and the amount of food intake was measured every week.

### 2.9. In Vivo Micro-CT

In vivo micro-CT (Skyscan1176, Brucker microCT, Kontich, Belgium) was used to measure the changes in the abdominal adipose tissue volume. The raw data from the abdomen (Lumbar 2–5) were acquired at the start and end of the experiment using micro-CT (Resolution: 18 μm, Voltage: 45 kV, Current: 200 μA, Filter: 1.0 mm Al filter, Exposure: 250 ms), and all animals were under respiratory anesthesia with isoflurane (Hanaph, Seoul, Republic of Korea) to minimize their movement during the scanning. The raw data were reconstructed into two-dimensional cross-sectional image slices using NRecon (Brucker micro-CT, Kontich, ver.1.6.9.3, Belgium). After that, the abdominal adipose tissue volume was evaluated by CT Analyzer (CT-AN ver.1.10.9.0, Brucker, Kontich, Belgium).

### 2.10. Body Weight and Liver Weight

In order to examine the changes in body weight during the experiment period, the weight of each animal was measured using a scale. To determine the weight of the liver, the liver was excised immediately after blood sampling and euthanasia, and then, the liver tissue was washed with saline and the surface moisture was removed to measure the weight.

### 2.11. Triglycerides in Serum

The mouse blood samples were collected by orbital puncture at the start and end of the experiment. The tubes were centrifuged at 2500 rpm for 10 min, and serum samples were stored at −80 °C. The triglyceride concentrations in serum (10 μL) was measured using the Triglyceride Quantification Assay Kit (ab65336, Abcam) according to the manufacturer’s protocols. In this assay, 2 μL of lipase was added in prepared samples and triglycerides are converted to free fatty acids and glycerol. Then, we added 50 μL of reaction mix containing triglyceride assay buffer, triglyceride probe and triglyceride enzyme mix into the sample. We mixed and incubated at RT for 60 min protected from light and then measured the output on a microplate reader (Epoch, BioTek Instruments, Winooski, VT, USA) at OD 570 nm.

### 2.12. Statistical Analysis

One-way ANOVA was performed to compare the cell viability and Oil Red O staining, and Tukey’s post hoc test was used. A paired *t*-test method was used to compare the value, including the body weight gain and the volume of abdominal adipose tissue, during the experimental period in each group. Results were presented as the means  ±  standard error (SE). Statistical analysis was performed using SPSS 25 (IBM SPSS Statistics, SPSS Inc., Chicago, IL, USA), and *p*  <  0.05 was considered statistically significant.

## 3. Results

### 3.1. Analysis of Cell Viability and Intracellular Lipid Droplet Formation According to MCS Application and Its Intensity

**Figure 1:**
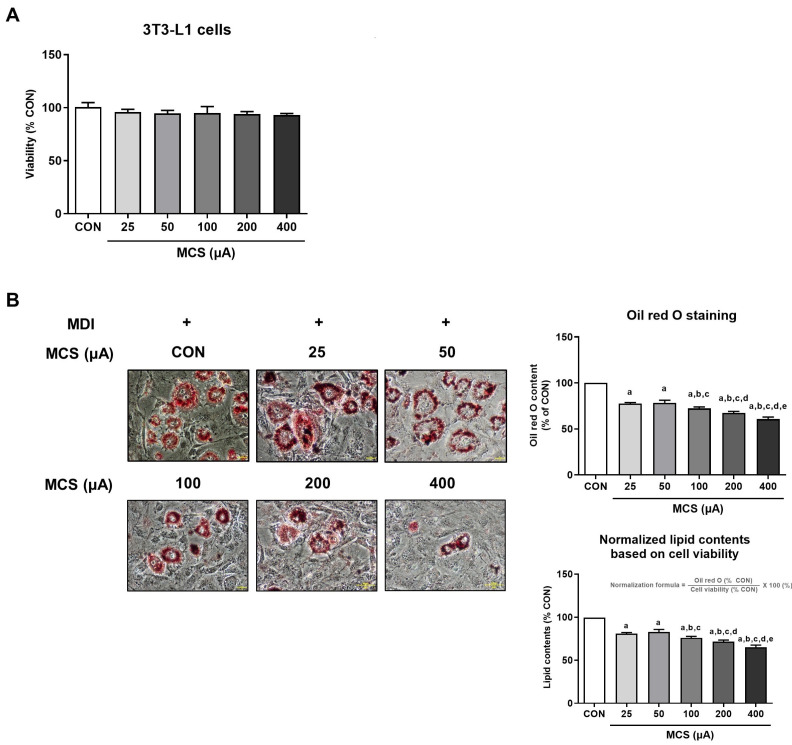
Cell viability and intracellular lipid droplet formation following the various intensities of MCS. (**A**) The cytotoxicity of 3T3-L1 cells was measured by the application of electrical stimulation (25, 50, 100, 200, and 400 μA). The values are expressed as the mean ± SE of independent experiments performed in triplicate. Values are shown as percentages of the control. (**B**) Microscopic images of Oil Red O stained 3T3-L1 cells and quantification of lipid droplet (magnification: 200X). Oil Red O staining results are also normalized in consideration of the cell viability. All values are expressed as the mean ± SE of independent experiments performed in triplicate and shown as percentages of the control. a: vs. CON, b: vs. 25 μA, c: vs. 50 μA, d: vs. 100 μA, e: vs. 200 μA, *p* < 0.05.

We measured the cell viability of 3T3-L1 cells according to the intensity of MCS to determine the effect of MCS on 3T3-L1 cells. As shown in Figure 1A, there was no significant change in MCS-applied groups compared to untreated cells (Figure 1A). In other words, cell viability was not affected by the intensity of MCS and also with or without the application. Oil Red O stain was performed to confirm the effect of MCS on lipid droplet formation in MDI-induced adipocytes. Adipocytes were pretreated with MCS (25, 50, 100, 200, and 400 μA) in a serum-free medium for 48 h. It was shown that lipid accumulation decreased in an intensity-dependent manner (Figure 1B). Although there was no significant difference in cell viability depending on the application of electrical stimulation, the lipid droplet accumulation rate was normalized in consideration of the cell viability. The value was calculated by the following formula: Oil Red O staining (% CON)/Cell viability (% CON) * 100. The results using the normalized values showed the same tendency as the results that were not normalized. These results indicate that MCS downregulates adipogenesis in MDI-induced 3T3-L1 cells without affecting cell viability.

### 3.2. Effect of MCS on Insulin Signaling Pathway

**Figure 2:**
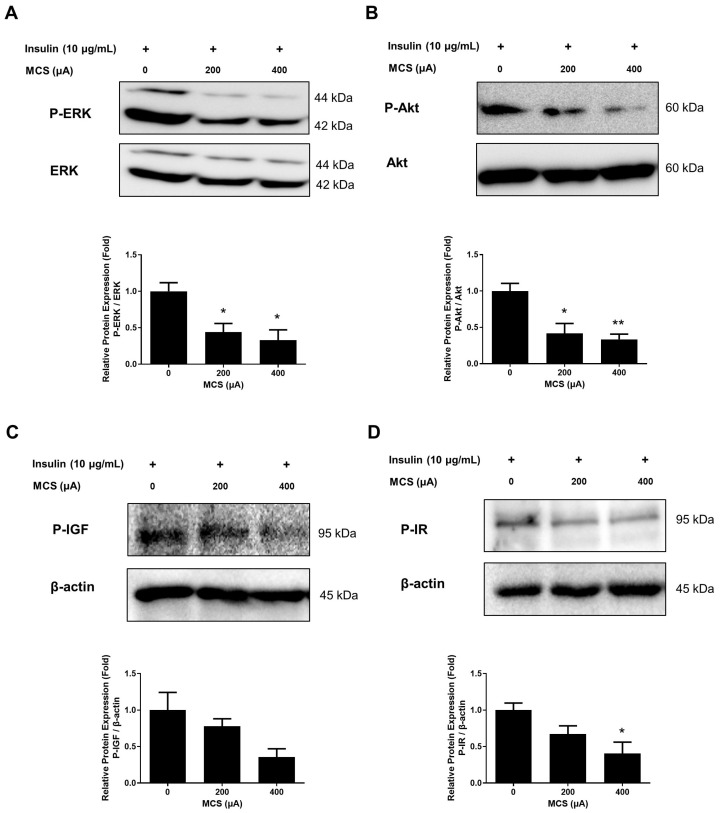
Effect of MCS on the expression of proteins regarding insulin signaling pathway: (**A**) The protein expression of p-ERK in 3T3-L1 cells. (**B**) The protein expression of p-Akt in 3T3-L1 cells. (**C**) The protein expression of p-IGF in 3T3-L1 cells. (**D**) The protein expression of p-IR in 3T3-L1 cells. The values are expressed as mean ± SE of independent experiments performed in triplicate. The graph represents the quantitative level of the proteins. * *p* < 0.05, ** *p* < 0.01 vs. CON group.

To investigate the effects of MCS on the adipogenesis of 3T3-L1 cells, phosphorylation of the ERK1/2, Akt, IGF, and IRβ signaling pathways was detected in MDI-induced 3T3-L1 cells. Phosphorylation of the ERK1/2 and Akt was increased in 3T3-L1 cells induced by insulin, whereas MCS significantly inhibited the phosphorylation of these induced by insulin in an intensity-dependent manner (Figure 2A,B). Especially, phosphorylation was significantly inhibited at concentrations of 400 µA. Additionally, the effect of MCS on the phosphorylation of IGF and IRβ, upper signals of MAPK and Akt, was examined. IRβ, a receptor protein subunit, showed increased expression levels, and IRβ phosphorylation was inhibited in MCS-treated cells (Figure 2D). In addition, MCS treatment decreased IGF phosphorylation in an intensity-dependent manner (Figure 2C). These results show that MCS can inhibit the phosphorylation of the insulin signaling pathway.

### 3.3. The Protein Expression of C/EBP-α and Nucleus Translocation of PPAR-γ in 3T3-L1 Adipocytes According to the Application of MCS

**Figure 3:**
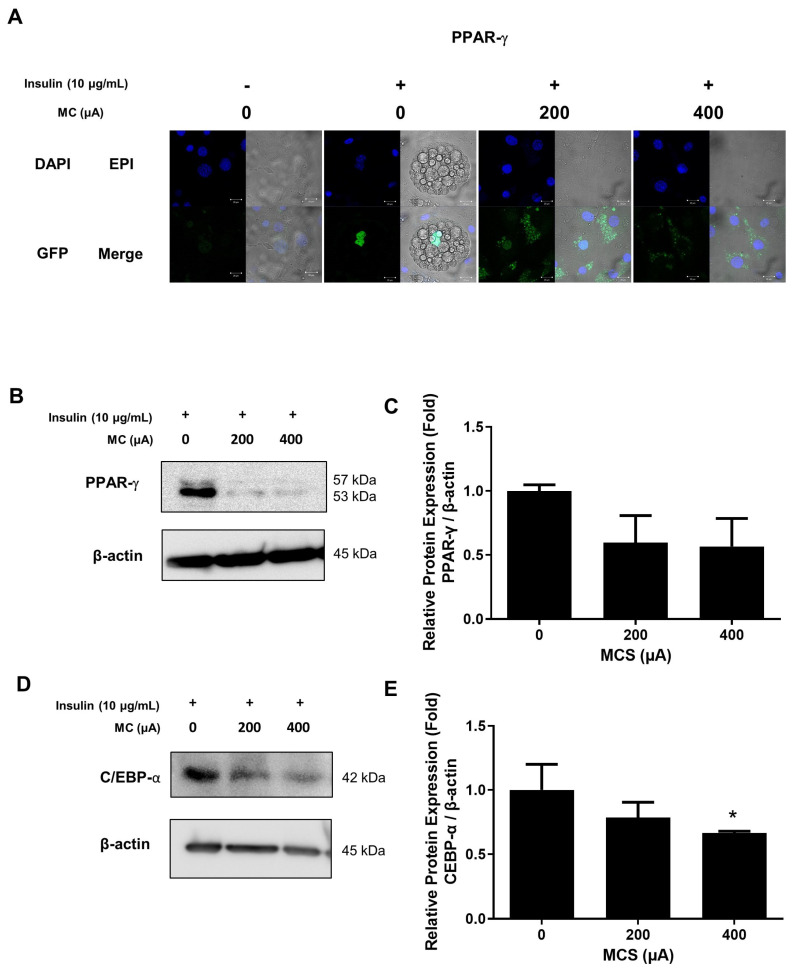
The protein expression of C/EBP-α and nucleus translocation of PPAR-γ in 3T3-L1 according to the application of MCS. (**A**) Representative images of confocal microscopy regarding PPAR-γ. Nuclei were visualized by DAPI (blue). Anti-PPAR-γ was visualized by GFP (green). (**B**) The protein expression of PPAR-γ in 3T3-L1 cells. (**C**) Relative protein expression level of PPAR-γ (**D**) The protein expression of C/EBP-α in 3T3-L1 cells. (**E**) Relative protein expression of C/EBP-α in 3T3-L1 cells. The values are expressed as mean ± SE of independent experiments performed in triplicate. The graph represents the quantitative level of the proteins. * *p* < 0.05 vs. CON group.

Adipocytes differentiation is induced by MDI cocktail, which is one of the conditions that caused the fibroblast-like preadipocytes to differentiate into round-shaped adipocytes [24]. Our result showed these morphological changes on the microscopy image as shown in Figure 3A. During 3T3-L1 adipogenesis, C/EBP-α and PPAR-γ are activated by insulin with FBS [25]. We investigated the changes in the expression level of transcription factors such as PPAR-γ according to the application of MCS during adipogenesis. In the result of confocal microscopy, PPAR-γ, identified as Alexa-488, significantly decreased protein expression in the nucleus (Figure 3A), and also the protein expression of PPAR-γ was significantly decreased with MCS application (Figure 3B). In addition, the PPAR-γ protein expression in the nucleus was remarkably decreased in 400 μA MCS- applied cells (Figure 3C). The protein expression level of C/EBP-α, another transcription factor related to adipogenesis, was also decreased by MCS (Figure 3D,E). These results showed the possibility that MCS can inhibit adipogenesis, so we conducted in vivo experiments to analyze changes in body weight, adipose tissue volume, and the protein expression level of C/EBP-α in white adipose tissue (WAT) following the application of MCS in *ob*/*ob* mice.

### 3.4. Changes in Body Weight and Food Intake and the Observation of the Appearance and the Protein Expression of C/EBP-α for the Epididymal Fat Pad

**Figure 4:**
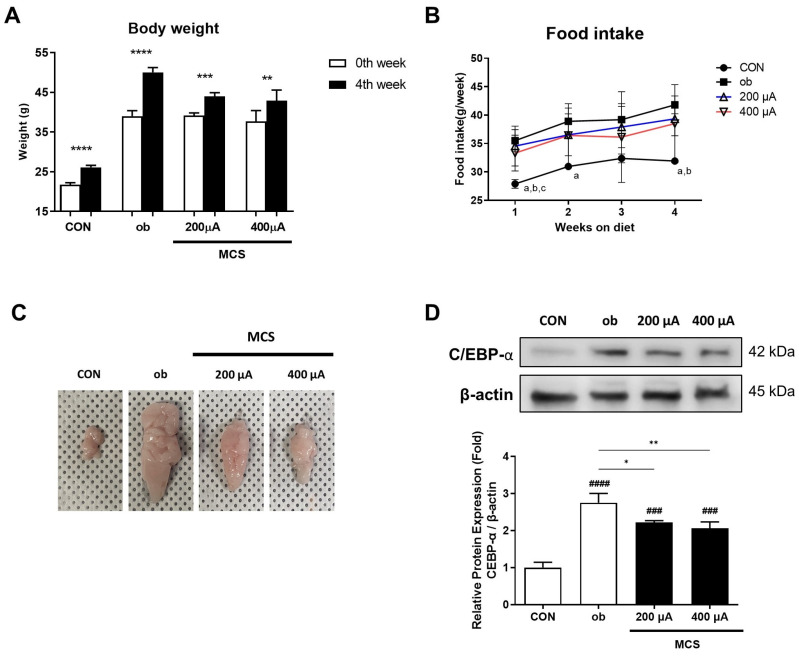
Changes in body weight and food intake and the observation of the appearance and the protein expression of C/EBP-α for the epididymal fat pad. (**A**) Body weights before and after the experiment. Values are expressed as mean ± SE. ** *p* < 0.01, *** *p* < 0.001, **** *p* < 0.0001 0th week vs. 4th week in each group. (**B**) Weekly food intake during the experimental periods. a: *p* < 0.05 vs. ob, b: *p* < 0.05 vs. 200 μA, c: *p* < 0.05 vs. 400 μA. (**C**) The appearance of the epididymal fat pad in each group. (**D**) The protein expression of C/EBP-α in epidydimal fat tissue. * *p* < 0.05, ** *p* < 0.01 vs. ob. ### *p* < 0.001, #### *p* < 0.0001 vs. CON group.

We examined the effect of MCS on weight loss and adipose tissue volume reduction through an *ob*/*ob* mouse model. Body weight increased significantly for 4 weeks in all groups (Figure 4A). However, the ob group increased by 28.3%, while the 200 and 400 μA increased by 12.3% and 13.8%, respectively, showing a small decrease. Average weekly food intake not significantly different between ob and MCS groups, as shown in Figure 4B. However, the food intake of the ob group remained higher than that of the CON group during most of the experimental periods (*p* < 0.05).

To observe changes in adipose tissue volume in animals depending on whether or not MCS was applied, visual observation was performed through epididymal fat pad removal (Figure 4C). The size of the epididymal fat pad showed the largest size in the ob group and showed a significantly smaller size in 200 and 400 μA. Moreover, the immunoblotting analysis for the epididymal fat pad was performed to examine the protein expression of C/EBP-α, which is the one of the adipogenic markers. As shown in Figure 4D, the expression level of C/EBP-α showed a tendency similar to the size of the epididymal fat pad. Compared to the CON group, all other groups showed significantly higher expression (*p* < 0.001), but the MCS-applied groups showed significantly lower expression compared to the ob group (200 μA, *p* < 0.01; 400 μA, *p* < 0.001).

These results implied that MCS is effective in inhibiting body weight gain and fat accumulation in animals.

### 3.5. Observation of Abdominal Adipose Tissue Using Micro-CT Analysis

**Figure 5:**
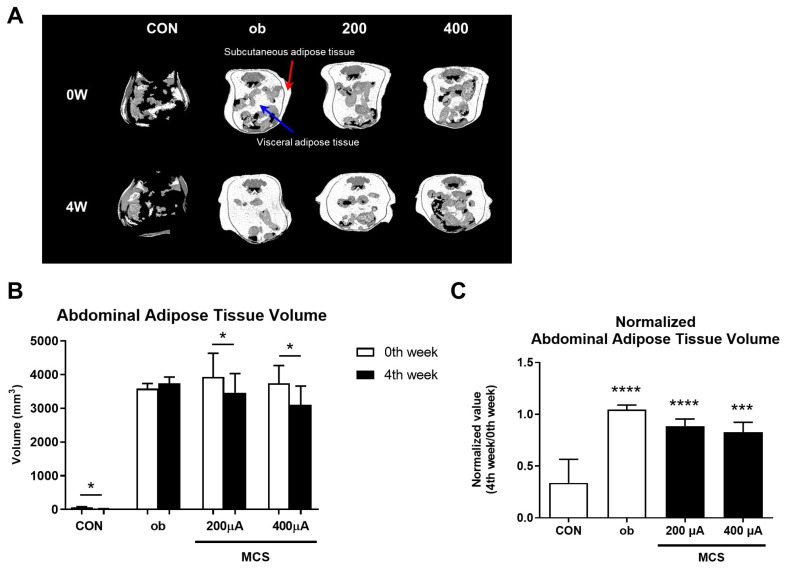
Micro-CT analysis results. (**A**) Representative 3D images of abdominal adipose tissue at the start and end of the experiment in each group. A red arrow indicates the region of subcutaneous adipose tissue. A blue arrow indicates the region of visceral adipose tissue. (**B**) The abdominal adipose tissue volume was calculated by Micro-CT images. Values are expressed as mean ± SE. * *p* < 0.05, 0th week vs. 4th week in each group. (**C**) The normalized value of abdominal adipose tissue volume based on the value in 0th week for comparing changes in abdominal adipose tissue volume between all groups. *** *p* < 0.001, **** *p* < 0.0001 vs. CON group.

Abdominal adipose tissue volume was calculated from micro-CT images. As shown in Figure 5A, 3D images of abdominal adipose tissue showed similar trends in terms of size as the Figure 4C. In particular, MCS groups tended to decrease both subcutaneous and visceral adipose tissues. As an additional analysis, the volume of abdominal adipose tissue was quantitatively calculated using the images scanned by micro-CT and shown in Figure 5B. As for the result of changes in abdominal adipose tissue volume in each group during the experimental period, the ob group showed a slight increase, whereas both the 200 and 400 μA showed a significant decrease (*p* < 0.05). There was no difference between the ob group and the MCS groups in the normalized value of abdominal fat tissue volume based on the 0th week value, but the significance level at 400 μA (*p* < 0.001) was lower than that of the ob and 200 μA groups (*p* < 0.0001) (Figure 5C).

### 3.6. Observation of Liver and Changes in Serum Triglycerides (TG) Levels

**Figure 6:**
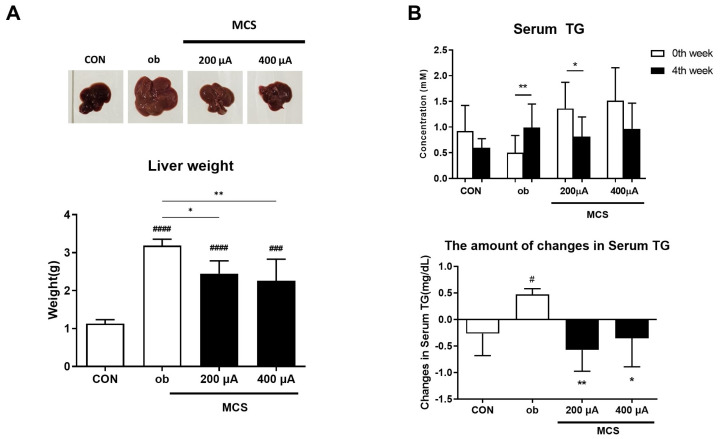
Observation of the liver and changes in serum triglycerides (TG) levels. (**A**) The appearance and weights of the liver following MCS. The values are expressed as mean ± SE. * *p* < 0.05, ** *p* < 0.01 vs. ob. ### *p* < 0.001, #### *p* < 0.0001 vs. CON group. (**B**) Changes in serum triglycerides (TG) levels at before and after the experiment. * *p* < 0.05, ** *p* < 0.01 0th week vs. 4th week in each group. The amount of changes in serum TG during the experimental periods also showed another graph. The values represent the amount of increase or decrease based on serum TG at the 0th week. The values are expressed as mean ± SE. * *p* < 0.05, ** *p* < 0.01 vs. ob. # *p* < 0.05 vs. CON group.

The appearance and weight of the liver were compared to evaluate whether MCS alleviates lipid deposition in the liver among the indicators of prolonged obesity. It can be seen that the liver of the ob group was enlarged in size and changed in color compared to CON (Figure 6A). The weight of the liver was also about three times that of CON, suggesting that the weight and size increased and the color changed due to lipid deposition. As another indicator, serum TG level, which is high in obesity, increased in ob while decreasing in the MCS groups (Figure 6B). Additionally, we compared the amount of changes in serum TG during experimental periods. The value in each group increased by 0.47 mM in ob, while it decreased by 0.57 mM and 0.35 mM in the 200 and 400 μA, respectively, based on the value at the onset of the experiment. There was a significant difference in ob when compared with CON, but both stimulation groups did not show a significant difference. These results suggest that MCS may be effective in alleviating obesity.

## 4. Discussion

In this study, we investigated the effect of MCS on adipogenesis in 3T3-L1 adipocytes and in the *ob*/*ob* mouse model. Adipogenesis is mostly referred as the process by which adipocytes differentiated from preadipocytes [26,27]. From previous studies, it has been reported that external stimulation such as magnetic field resonance and ultraviolet radiation is known to affect the obesity. There was a report that the fat combustion rate significantly increased when water-filtered infrared-A (wIRA) treatment and exercise were performed together [28]. Ultraviolet radiation (UVR) helps synthesize vitamin D. Adipocytes express vitamin D receptors, and they are responsible for the active vitamin D metabolite. Consequently, obesity and vitamin D deficiency are deeply related [29,30]. The role of magnetic field resonance is also being studied in obesity [31]. Our previous studies have reported on regulating the adipogenesis signaling process by using MCS via the β-catenin signaling pathway. In this study, we focused on whether MCS can regulate the insulin signaling pathways [17]. To observe the various proteins that are associated with insulin signaling pathways, we conducted immunoblotting.

Our study showed that MCS reduces lipid droplets in 3T3-L1 cells through Oil Red O staining [32,33]. The lipid droplets are produced and maintained by various factors such as cell death inducing DFFA-like effector c (CIDEC), LIPE, and perilipin 1 (PLIN1). Further studies are needed regarding whether MCS actually modulates those factors [34,35]. 

Adipocytes differentiation is mediated by the phosphorylation of insulin receptor pathways and MDI stimulation induction [36,37]. In other studies, it is suggested that modulating the cell cycle is an efficient strategy to reduce lipid accumulation in the multi-clonal expansion (MCE) period [38,39]. We have reported in previous studies that MCS contributed to the cell cycle progression of HFDPC cells at 25 μA and 50 μA concentrations for hair growth [40]. However, further studies are needed regarding whether MCS affects adipose cell cycle-related proteins.

Insulin receptor signals initiated by the phosphorylation of insulin receptors subunit and lipid accumulation is mediated through the insulin signaling pathway in 3T3-L1 cells [41]. Insulin receptors and insulin-like growth factor receptors are phosphorylated by insulin treatment [42]. To confirm the effect of MCS on inhibiting lipid accumulation, it was examined whether insulin signaling can be modulated by the application of MCS. The phosphorylation of insulin signaling pathway proteins such as IR, IGF, ERK, and Akt was inhibited by MCS. We also confirmed that MCS leads to a decrease in the expressions of C/EBP-α and PPAR-γ, which are known as transcription factors in the adipogenesis. In other studies, a reduction in transcription factors has been mentioned as an efficient strategy for inhibiting lipid accumulation [25,43]. It supported the results of our study.

In order to examine the effects of MCS on inhibiting adipogenesis, we measured various indicators related to obesity by using an *ob*/*ob* mouse model. The *ob*/*ob* obese mouse model is an animal model that causes genetic obesity due to leptin deficiency and excessive food intake [44]. For these reason, it has been mainly used in the research on type 2 diabetes or obesity [45]. Our results revealed that both body weight gain and abdominal adipose tissue volume were decreased in all MCS groups compared to the ob group. In particular, there was no significant difference in the comparison of food intake between the MCS groups and the ob group, but the abdominal adipose tissue volume increased in the ob group while it decreased in all MCS groups (Figure 4B and Figure 5B,C). These results support that MCS effectively alleviates obesity in *ob*/*ob* mice. 

Moreover, both the epididymal fat pad and liver were excised at the end of the experiments to observe the differences in the appearance of these tissues. The size of the epididymal fat pad, which consists mainly of WAT in animal models, was noticeably smaller in all MCS groups than in the ob group. We also investigated the protein expression of C/EBP-α in epididymal fat lysates. The expression level was significantly decreased in all MCS groups, supporting the result of using 3T3-L1 cells. According to liver tissue observations, the ob group showed an enlarged liver. On the other hand, a relatively small size and weight were shown in the MCS groups. These results might be related to serum TG level, as shown in Figure 5B. Dyslipidemia is strongly associated with obesity and metabolic syndrome [46]. When triglycerides are hydrolyzed, they are divided into free fatty acids and glycerol, which could be used as energy sources in the absence of sugars [47,48]. Contrastingly, excess free fatty acid could be converted into triglycerides through lipid synthesis processes in liver tissue [49]. We confirmed that MCS reduced the concentration of triglycerides in serum and suppressed liver hypertrophy in *ob*/*ob* mice. Therefore, the regulation of triglycerides by MCS could also contribute to the mechanisms for the regulation of lipogenesis or non-alcoholic fatty liver disease. 

Taken together, the lipid accumulation was suppressed through MCS in 3T3-L1 adipocytes and *ob*/*ob* obese mice. Recent studies have mentioned that lipid accumulation may be reduced due to a decrease in transcription factor expression such as C/EBP-α and PPAR-γ [50,51]. Our results also confirmed the inhibition of lipid metabolic-related transcription factors, C/EBP-α and PPAR-γ, in 3T3-L1 adipocytes. The reduction in the volume of WAT through MCS could contribute to the prevention of obesity and metabolic syndrome in the *ob*/*ob* obese model.

## 5. Conclusions

In this study, the effect of MCS was confirmed through insulin signaling pathway regulation in the lipid accumulation process. MCS induced downregulation in the phosphorylation of downstream signals such as Akt and ERK through insulin signaling pathways. The expression levels of C/EBP-α and PPAR-γ are decreased in 3T3-L1 adipocytes, and the expression level of C/EBP-α was also decreased in animal adipose tissue. In particular, MCS reduced body weight gain and decreased serum TG in *ob*/*ob* obese mouse model. It also significantly reduced the volume of liver and adipose tissue. Based on our results, we suggest that MCS might be a useful method for the regulation of insulin signaling pathways to inhibit the adipogenesis process.

## Data Availability

Not applicable.
